# Resuming anti-TNF therapy after development of miliary tuberculosis in Behcet’s disease-related uveitis: a case report

**DOI:** 10.1186/s12348-023-00375-w

**Published:** 2023-11-28

**Authors:** Chika Toriu, Kinya Tsubota, Yoshihiko Usui, Hiroshi Goto

**Affiliations:** Department of Ophthalmology, Tokyo Medical University, 6-7-1 Nishishinjuku, Shinjuku-Ku, Tokyo, 160-0023 Japan

## Abstract

**Purpose:**

There is no consensus concerning restarting anti-tumour necrosis factor (TNF)-α therapy for uveitis after treatment for active tuberculosis (TB). We report a case of Behcet disease (BD) in which treatment with TNF inhibitor was successfully resumed after treatment for miliary TB.

**Case report:**

A 48-year-old Japanese male was treated for uveitis of unknown aetiology in the left eye at a general ophthalmology clinic. He was referred to Department of Ophthalmology, Tokyo Medical University Hospital because of macula oedema (ME) not responding to prednisolone (PSL) 20 mg. BD was diagnosed based on fluorescein angiographic findings of diffuse retinal vasculitis characteristic of BD, recurrent oral aphthous ulcer, erythema nodosum-like rash in his legs, and HLA-A26 positivity. After a screening test, adalimumab (ADA) was started as steroid-sparing therapy. Eight months after starting ADA, the patient was diagnosed with miliary TB. ADA and PSL were discontinued immediately due to TB. Anti-TB treatment was completed after 6 months based on clinical improvement, although T-SPOT.TB was still positive. Infliximab with isoniazid was started due to relapse of ME, worsened vitreous haze, and worsened visual acuity in his left eye. Subsequently, his ocular symptoms subsided and there was no relapse of TB.

**Conclusion:**

This case suggests that in patients with BD who have discontinued anti-TNF therapy due to miliary TB, restarting anti-TNF therapy may be a therapeutic option after TB has been treated appropriately with careful monitoring for relapse.

Uveitis may cause acute visual deterioration, and have diverse aetiologies including Bechet disease (BD) [1]. BD is a relatively frequent cause of uveitis in Japan, constituting 6.2 to 7.6% of all uveitis [1]. The advent of tumour necrosis factor (TNF)-α inhibitors such as infliximab (IFX) and adalimumab (ADA) has significantly improved visual acuity in non-infectious uveitis including BD [2–6]. In Japan, IFX has been approved since 2007 for the treatment of refractory uveitis in BD, and ADA has been approved since 2016 for the treatment of refractory non-infectious uveitis including BD [7]. While TNF inhibitors have been evaluated for their efficacy in the treatment of BD, many adverse events such as infections, infusion reactions and malignancy have also been reported [3, 8, 9]. An international study on the long-term outcomes of ADA in patients with non-infectious uveitis showed that infection was the most common adverse event occurring in 65% of patients, including 4.7% of tuberculosis (TB) [8]. Another study of long-term safety of ADA in Japanese patients with non-infectious uveitis also found that infection was the most common adverse drug reaction (8.4%), including 1.6% of TB [9]. If TB infection occurs during TNF inhibitor therapy, treatment should be discontinued. In addition, it is important to initiate anti-TB medications immediately.

TB treatment usually requires a period of 3 to 9 months, and TNF inhibitors should be suspended during this treatment period [10]. It is generally accepted that TNF inhibitors can be restarted after TB treatment if the TB treatment is completed successfully [11, 12]. However, there are no detailed reports of the clinical course of ophthalmic diseases in which treatment with TNF inhibitor that was discontinued due to TB was resumed. We report a case of Behcet's disease in which treatment with a TNF inhibitor was resumed successfully after discontinuation due to development of miliary TB.

## Case report

**Fig. 1:**
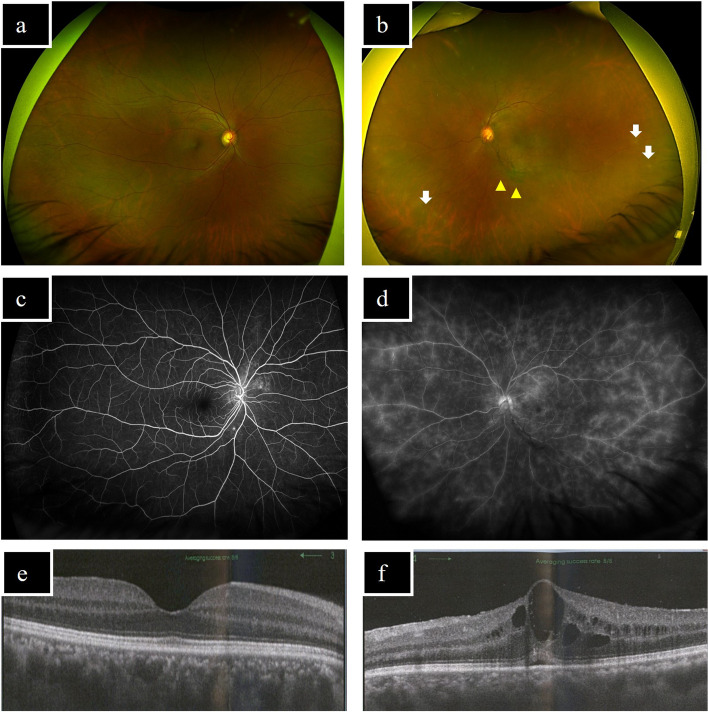
Imaging findings at presentation to our facility. (**a**, **b**): Fundus photographs. **a** No remarkable inflammatory changes in the retina are found in right eye. **b** Diffuse vitreous haze (yellow arrow-head) and mild exudates in peripheral retina (white arrow) are observed in left eye. (**c**, **d**): Fluorescein angiography images. **c** No remarkable findings are observed in right eye. **d** Diffuse vascular leakage at the posterior pole and periphery retina are observed in left eye. (**e**, **f**) OCT images. **e** No remarkable inflammatory changes at the macula are found in right eye. **f** Macular oedema and mild subretinal fluid are observed in left eye

A 48-year-old Japanese male was treated for uveitis of unknown aetiology in the left eye at a general ophthalmology clinic. Prednisolone (PSL) 40 mg/day was started due to the development of macula oedema (ME) in his left eye. Although ME improved temporarily, tapering of PSL to 20 mg/day resulted in relapse of ME. He then complained of blurred vision in the left eye due to recurrence of intraocular inflammation and was referred by the local ophthalmologist to Department of Ophthalmology, Tokyo Medical University Hospital. He has no medical history. At presentation at our facility, best-corrected visual acuity (BCVA) was 20/12.5 in the right and 24/20 in the left eye, and intraocular pressure was normal. Slit-lamp examination revealed 1 + cells in anterior chamber in left eye; fundus photographs showed diffuse vitreous haze and mild exudates in peripheral retina in left eye; fluorescent angiography depicted significant retinal vasculitis characteristic of Bechet’s disease at the posterior pole and periphery retina in left eye; and OCT confirmed ME in left eye (Fig. 1). Peripheral blood test showed elevated white blood cell count, elevated percent neutrophils, elevated LDH, positive for HLA-A26, negative for HLA- B51, negative tuberculin skin test (TST) and negative T-SPOT.TB test (Table 1). He was diagnosed with incomplete type of Bechet disease based on the ophthalmological findings, recurrent oral ulcer, erythema nodosum-like rash in his legs, and HLA-A26 positivity. After a screening test, ADA was started as steroid-sparing therapy. Following the initiation of ADA, ME improved gradually and tapering of PSL to 2 mg/day was achieved, resulting in favourable outcome.

**Table 1:** Laboratory findings

Tests			Tests		
White blood cells	9,900	/µl	IgG	1,126	mg/dl
Neutrophil	75.9	%	IgA	195	mg/dl
Basophil	0.5	%	IgM	56	mg/dl
Eosinophil	0.3	%	ANA	< 40	
Lymphocyte	19	%	Rheumatoid factor	3.6	IU/ml
Red blood cell	475 × 10^4^	/µl	ACE	13.6	U/L
Hemoglobin	14.7	g/dl	Anti-streptolysin-O	44	IU/ml
Hematocrit	43.4	%	Anti-toxoplasma IgG	< 3	
Platelet	234 × 10^4^	/µl	CMV antigen (C7HRP)	negative	
AST	23	IU/L	RPR	negative	
ALT	30	IU/L	TPLA	negative	
LDH	216	IU/L	HBs	negative	
Glucose	97	mg/dl	HCV	negative	
Blood urea nitrogen	11.6	mg/dl	HLA-B51	negative	
Creatinine	0.7	mg/dl	HLA-A26	positive	
C-reactive protein	0.1	mg/dl	TST	negative	
sIL-2R	483	U/ml	T-SPOT. TB	negative	
CH50	58	U/ml			

*AST* Aspartate aminotransferase, *ALT* Alanine aminotransferase, *LDH* Lactate dehydrogenase, *sIL-2R* Soluble interleukin-2 receptor, *CH50* 50% hemolytic complement activity, *IgG* Immunoglobulin G, *IgA* Immunoglobulin A, *IgM* Immunoglobulin M, *ANA* Antinuclear antibody, *ACE* Angiotensin-converting enzyme, *CMV* Cytomegalovirus, *RPR* Rapid Plasma Reagin, *TPLA* Treponema pallidum latex agglutination, *HBs* Hepatitis B surface antigen, *HCV* Hepatitis C virus antibody, *HLA* Human Leukocyte Antigen, *TST* Tuberculin skin test

**Fig. 2:**
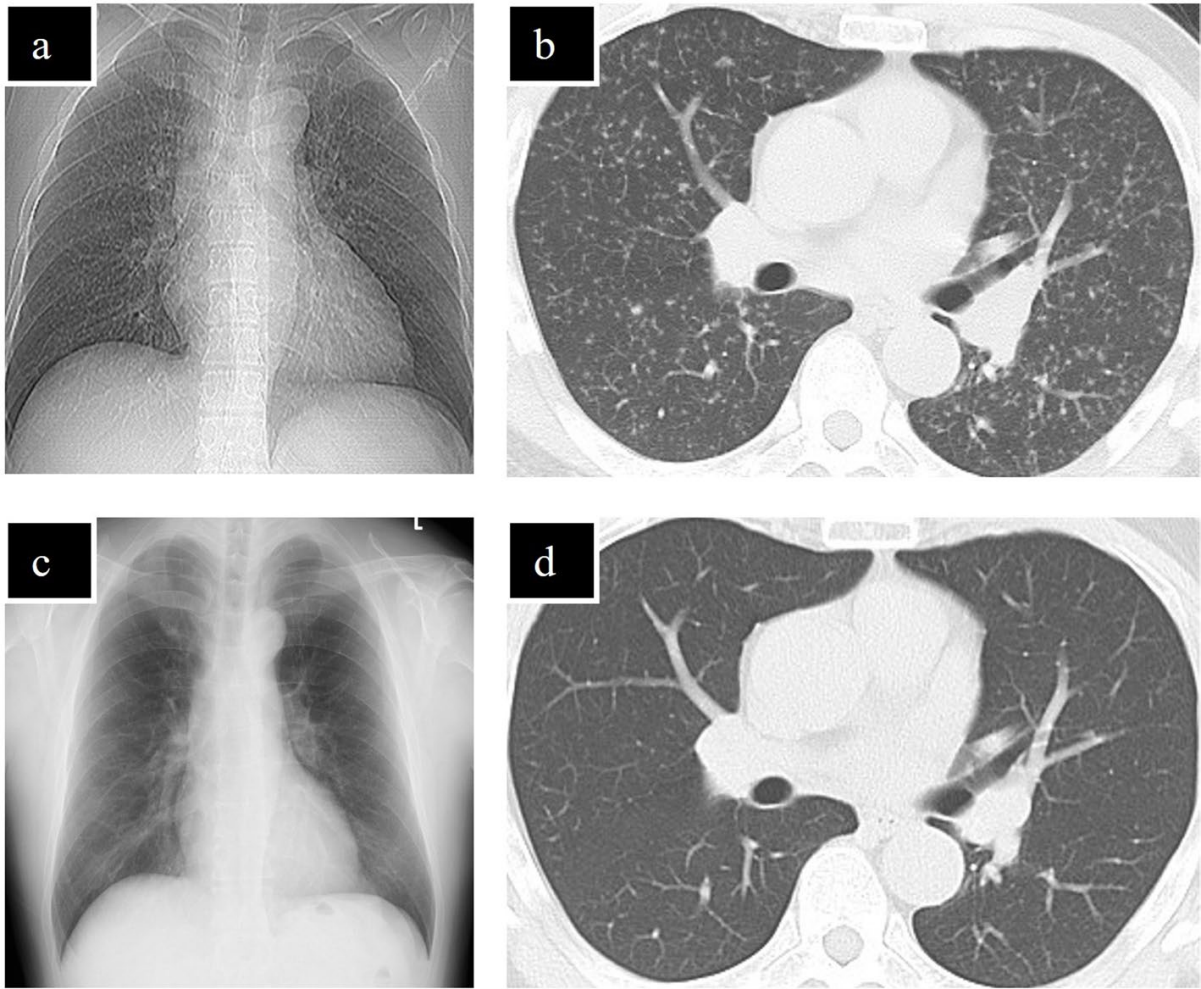
Chest X-ray and CT before and after treatment for miliary TB. Chest X-ray (**a**) and CT (**b**) images before treatment reveal multiple diffuse granular shadows in bilateral lungs and a lesion near the pleura with suspected hematogenous distribution. Chest X-ray (**c**) and CT (d) images after treatment show improvement of multiple diffuse granular shadows in bilateral lungs

**Fig. 3:**
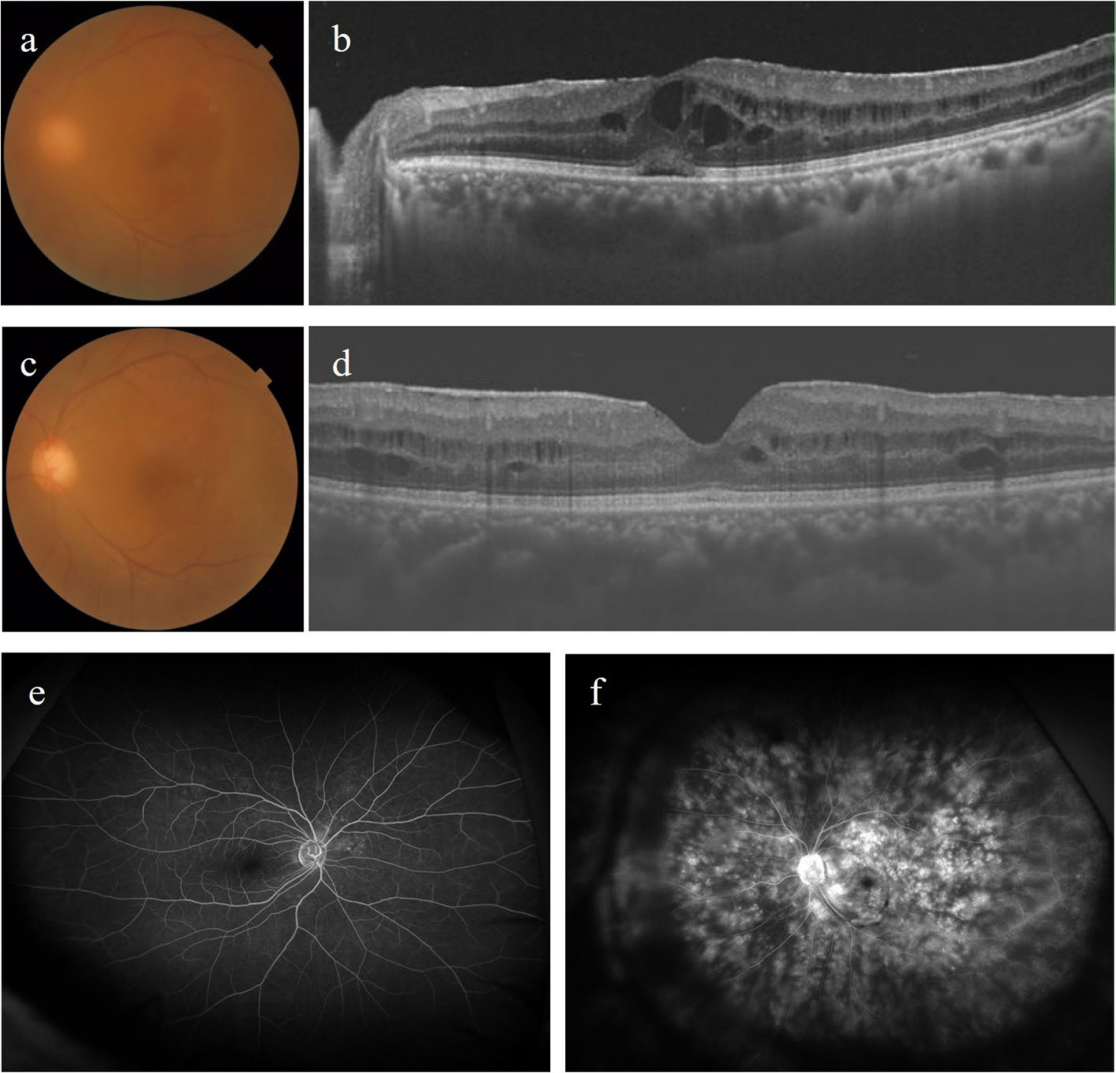
Imaging findings before and after resuming TNF inhibitor. After discontinuation of TNF inhibitor (adalimumab), fundus photograph (**a**) and OCT image (**b**) show worsening of vitreous opacity and macular oedema in left eye. After resuming TNF inhibitor (infliximab), fundus photograph (**c**) and OCT image (**d**) show improvement of vitreous opacity and macular oedema. After resuming TNF inhibitor (infliximab), fluorescein angiography shows no remarkable findings in right eye (**e**), but depicts diffuse vascular leakage at the posterior pole and periphery retina in left eye (**f**)

However, 8 months after starting ADA, the patient developed general malaise. Peripheral blood examination was positive for T-SPOT.TB at this time, and chest X-ray and computed tomography (CT) showed granular shadows in bilateral lungs (Fig. 2a, b). He was diagnosed with miliary TB by a respiratory physician. ADA and PSL were discontinued immediately, and 4-drug regimen for miliary TB consisting of isoniazid (300 mg/day), rifampicin (450 mg/day), ethambutol (750 mg/day) and pyrazinamide (dose titrated from 0.8 g/day to 1.5 g/day) was started. The TB treatment was completed 6 months later based on clinical improvement (Fig. 2c, d), although T-SPOT.TB was still positive. During the period when ADA was discontinued, the patient received adjunctive therapy with betamethasone eye drops and sub-Tenon's injection of triamcinolone acetonide to manage the condition. However, the efficacy of these treatments approach was limited to control ME (Fig. 3a, b), resulting in decreased BCVA in the left eye (10/20) and gradual elevation of intraocular pressure due to topical administration of steroid. Therefore, a decision was made to restart TNF inhibitor. Although miliary TB treatment had been completed, the patient still tested positive for T-SPOT.TB, necessitating initiation of infliximab (IFX) in combination with isoniazid (300 mg/day) to address the condition. Following the initiation of IFX, subsequent fluorescein angiography demonstrated more severe vascular leakage compared to the initial examination. However, improvements of ME, vitreous opacity (Fig. 3c-f) and BCVA in the left eye (24/20) were observed. Furthermore, there was no TB relapse during the course of treatment after starting IFX for 3 years.

## Discussion

Bechet’s disease is a multisystemic inflammatory disorder characterized by diverse manifestations including recurrent oral and genital ulcers, ocular inflammation, and skin lesions. TNF inhibitors have emerged as a therapeutic option for refractory BD, providing symptom relief and reducing disease activity [2–7]. Despite the benefits of TNF inhibitors in BD, the risk of TB is a most significant concern, especially in endemic country including Japan. TNF inhibitors suppress the immune response, potentially increasing the risk of reactivation of latent TB infection or new TB infection. Several case reports and observational studies have reported TB as an adverse drug reaction in patients receiving TNF inhibitors for BD treatment [8, 9]. On the other hand, restarting TNF inhibitors in patients who developed TB as a complication of previous TNF inhibitor use has been reported in fields other than ophthalmology [11, 12]. In one study, of 16 patients who resumed biological agents after development of TB under anti-TNF therapy, one patient with Behcet's disease resumed TNF inhibitors [13], but there are no reports detailing the clinical course of TNF inhibitor resumption after completion of TB treatment in patients with Behcet's disease or other refractory uveitis.

Compared to other immune suppressive therapy, TNF inhibitors have a higher risk of TB [14]. Details of the mechanism by which TNF inhibitors increase the risk of developing TB are not fully understood. Monocyte-derived macrophages (MDM) are activated by TNF-α secreted by TB-infected MDM [15]. The activated macrophages kill TB or isolate it by granuloma formation. TNF gene-deficient mice that do not form granulomas are highly susceptible to TB, [16] suggesting that TNF inhibitors are associated with a higher risk of developing TB. Moreover, membrane TNF expressed by activated macrophages and T lymphocytes is essential for protection against TB infection [17, 18], as a difference in the incidence of TB has been observed between infliximab (an anti-TNF monoclonal antibody) and etanercept (soluble TNF receptor fusion molecule) [19, 20]. Studies have reported that TNF inhibitors suppress the proliferation of TB-specific T cells and the production of IFN and IL-1, which in turn suppress TB-specific T cells and increase the risk of developing TB [18, 21, 22]. Therefore, TB screening before starting TNF inhibitor is necessary, using chest X-ray, TST or interferon-γ release assays (IGRAs) such as QuantiFERON-TB Gold In-Tube (QFT-GIT) and T-SPOT.TB [23, 24]. 

False negative result of TST may occur due to cutaneous allergy, recent TB infection, recent live virus vaccination (measles, mumps, polio), or improper test administration [25]. Advanced age and low number of lymphocytes are risk factors of false negative result of IGRAs [26]. While some reports indicate an association between steroid use and negative or indeterminate QFT-GIT, [27, 28] others show no statistically significant association between steroid use and negative or indeterminate T-SPOT.TB [28, 29]. Our patient was receiving PSL before being referred to our hospital. We cannot rule out the possibility that use of PSL in the present case may have resulted in false negative result in TST and T-SPOT.TB at presentation. On the other hand, the number of TB cases is high in the Asian and African regions. Unfortunately, the incidence of TB in Japan is not low [30]. Health care workers in contact with patients are at higher risk of TB infection than those with no patient contact [31]. Our patient was a radiologist. There is a possibility that our patient had frequent contact with TB-positive patients in the course of his work. It is unclear whether TB was not diagnosed at presentation to our facility because of false-negative results due to PSL, or whether he developed TB through contact with a TB patient after the initiation of ADA.

Patients with active TB that developed after TNF inhibitor therapy should stop all immunosuppressive medications and immediately receive appropriate treatment for TB [32, 33]. Resuming TNF inhibitor is recommended after 2 to 4 weeks of isoniazid if there are no signs or symptoms of active TB [12]. If active TB is present, restarting TNF inhibitors is recommended after 3–4 months of TB treatment [34]. Six of 13 patients who developed TB during TNF inhibitor therapy had no TB recurrence by resuming TNF inhibitor after TB treatment; four restarted TNF inhibitor within 2 months after TB treatment and two restarted after completion of TB treatment [11]. Therefore, resuming TNF inhibitors after completion of TB treatment is considered safe. While early resumption of TNF inhibitors has been shown to prevent worsening of inflammatory disease, there is a report of TB recurrence in 2 of 30 patients who resumed TNF inhibitors after TB treatment [35].

In the current case, during the period when ADA was discontinued, BCVA, vitreous opacity and ME in the left eye worsened and intraocular pressure increased despite sub-Tenon's injection of triamcinolone acetonide. Fluorescent leakage may have been mildly reduced by PSL at the time of presentation, but fluorescent funduscopic examination after discontinuing of TNF inhibitor showed a higher degree of fluorescent leakage than that at presentation. Worsening of fluorescent leakage compared to that at presentation is likely due to barrier damage in retinal vessels as a result of prolonged period of no TNF inhibitor treatment, or fluorescence leakage at the initial examination may have been slightly masked because the patient was on prednisolone at presentation. Nevertheless, additional anti-inflammatory treatment was essential in this case. Therefore, the clinical decision was to restart anti-TNF therapy using IFX because the patient needed immediate improvement of inflammation and oral PSL was not appropriate due to Behcet's disease. Among TNF inhibitors, etanercept has been reported to have the lowest risk of TB reactivation, [36] but there are reported cases of BD with TB relapsing while on etanercept [13]. In our patient, we selected infliximab to resume anti-TNF therapy because the patient needed immediate improvement. On the other hand, TB has high recurrence rate, and TB recurrence in 2 of 30 patients who resumed TNF inhibitors after TB treatment has been reported [35]. In our patient, isoniazid (300 mg/day) was given as prophylaxis for TB recurrence, because TSPOT.TB remained positive even after completion of TB treatment and the patient was likely to be exposed to TB patients due to his occupation as a radiologist. Isoniazid prophylaxis is scheduled to be continued at least until T-SPOT.TB is confirmed negative. After resuming TNF inhibitor, the patient had a good clinical course with improving BCVA, vitreous opacity, ME in the left eye and no recurrence of TB. Currently, there are no clear criteria for resuming TNF inhibitors, as it depends on the underlying disease, severity of TB, and availability of other alternative therapies. There is a need to accumulate case reports of patients who were able to resume TNF inhibitors, such as the present case, and to develop resumption criteria.

## Data Availability

Data sharing is not applicable to this article as no datasets were generated or analysed during the current study.

## Abbreviations

TNF: Anti-tumour necrosis factor
TB: Tuberculosis
BD: Behcet disease
ME: Macula oedema
PSL: Prednisolone
ADA: Adalimumab
BCVA: Best-corrected visual acuity
TST: Tuberculin skin testing
CT: Computed tomography
MDM: Monocyte-derived macrophages
IGRAs: Interferon-γ release assays
QFT-GIT: QuantiFERON-TB gold in-Tube

## References

[1] Kunimi K, Usui Y, Tsubota K (2021). Changes in etiology of uveitis in a single center in Japan. Ocul Immunol Inflamm.

[2] Okada AA, Goto H, Ohno S, Mochizuki M Ocular Behçet's Disease Research Group Of Japan (2012). Multicenter study of infliximab for refractory uveoretinitis in Behçet disease. Arch Ophthalmol.

[3] Takeuchi M, Kezuka T, Sugita S (2014). Evaluation of the long-term efficacy and safety of infliximab treatment for uveitis in Behçet’s disease: a multicenter study. Ophthalmology.

[4] Jaffe GJ, Dick AD, Brézin AP (2016). Adalimumab in patients with active noninfectious uveitis. N Engl J Med.

[5] Sener H, Evereklioglu C, Horozoglu F, Gunay Sener AB (2023). Efficacy and safety of adalimumab in patients with Behçet Uveitis: a systematic review and meta-analysis. Ocul Immunol Inflamm. 1–9. Advance online publication. 10.1080/09273948.2022.2157288 10.1080/09273948.2022.2157288 36625549

[6] Kunimi K, Usui Y, Asakage M (2022). Anti-TNF-α therapy for refractory uveitis associated with Behçet's syndrome and sarcoidosis: a single center study of 131 patients. Ocul Immunol Inflamm.

[7] Maruyama K (2019). Current standardized therapeutic approach for uveitis in Japan. Immunol Med.

[8] Suhler EB, Jaffe GJ, Fortin E (2021). Long-term safety and efficacy of adalimumab in patients with noninfectious intermediate uveitis, posterior uveitis, or panuveitis. Ophthalmology.

[9] Namba K, Kaburaki T, Tsuruga H (2022). Long-term safety and effectiveness of adalimumab in Japanese patients with noninfectious intermediate, posterior, or panuveitis: post-marketing surveillance of 251 patients. Ophthalmol Ther.

[10] Theis VS, Rhodes JM (2008). Review article: minimizing tuberculosis during anti-tumour necrosis factor-alpha treatment of inflammatory bowel disease. Aliment Pharmacol Ther.

[11] Kim YJ, Kim YG, Shim TS (2014). Safety of resuming tumour necrosis factor inhibitors in patients who developed tuberculosis as a complication of previous TNF inhibitors. Rheumatology (Oxford).

[12] Swoger JM, Regueiro M (2014). Stopping, continuing, or restarting immunomodulators and biologics when an infection or malignancy develops. Inflamm Bowel Dis.

[13] Ozguler Y, Hatemi G, Ugurlu S (2016). Re-initiation of biologics after the development of tuberculosis under anti-TNF therapy. Rheumatol Int.

[14] Evangelatos G, Koulouri V, Iliopoulos A et al (2020) Tuberculosis and targeted synthetic or biologic DMARDs, beyond tumor necrosis factor inhibitors. Ther Adv Musculoskelet Dis. 12:1759720X20930116. 10.1177/1759720X20930116 10.1177/1759720X20930116 PMC7309385 32612710

[15] Giacomini E, Iona E, Ferroni L (2001). Infection of human macrophages and dendritic cells with Mycobacterium tuberculosis induces a differential cytokine gene expression that modulates T cell response. J Immunol.

[16] Bean AG, Roach DR, Briscoe H (1999). Structural deficiencies in granuloma formation in TNF gene-targeted mice underlie the heightened susceptibility to aerosol Mycobacterium tuberculosis infection, which is not compensated for by lymphotoxin. J Immunol.

[17] Plessner HL, Lin PL, Kohno T (2007). Neutralization of tumor necrosis factor (TNF) by antibody but not TNF receptor fusion molecule exacerbates chronic murine tuberculosis. J Infect Dis.

[18] Hamdi H, Mariette X, Godot V (2006). Inhibition of anti-tuberculosis T-lymphocyte function with tumour necrosis factor antagonists. Arthritis Res Ther.

[19] Tubach F, Salmon D, Ravaud P (2009). Risk of tuberculosis is higher with anti-tumor necrosis factor monoclonal antibody therapy than with soluble tumor necrosis factor receptor therapy: The three-year prospective French Research Axed on Tolerance of Biotherapies registry. Arthritis Rheum.

[20] Cantini F, Niccoli L, Goletti D (2014). Adalimumab, etanercept, infliximab, and the risk of tuberculosis: data from clinical trials, national registries, and postmarketing surveillance. J Rheumatol Suppl.

[21] Saliu OY, Sofer C, Stein DS (2006). Tumor-necrosis-factor blockers: differential effects on mycobacterial immunity. J Infect Dis.

[22] Wang J, van Dongen H, Scherer HU (2008). Suppressor activity among CD4+, CD25++ T cells is discriminated by membrane-bound tumor necrosis factor alpha. Arthritis Rheum.

[23] Hanta I, Ozbek S, Kuleci S (2008). The evaluation of latent tuberculosis in rheumatologic diseases for anti-TNF therapy: experience with 192 patients. Clin Rheumatol.

[24] Jung YJ, Woo HI, Jeon K (2015). The significance of sensitive interferon gamma release assays for diagnosis of latent tuberculosis infection in patients receiving tumor necrosis factor-α antagonist therapy. PLoS ONE.

[25] Huebner RE, Schein MF, Bass JB (1993). The tuberculin skin test. Clin Infect Dis.

[26] Yamasue M, Komiya K, Usagawa Y (2020). Factors associated with false negative interferon-γ release assay results in patients with tuberculosis: A systematic review with meta-analysis. Sci Rep.

[27] Liao CH, Lai CC, Tan CK (2009). False-negative results by enzyme-linked immunospot assay for interferon-gamma among patients with culture-confirmed tuberculosis. J Infect.

[28] Vassilopoulos D, Tsikrika S, Hatzara C (2011). Comparison of two gamma interferon release assays and tuberculin skin testing for tuberculosis screening in a cohort of patients with rheumatic diseases starting anti-tumor necrosis factor therapy. Clin Vaccine Immunol.

[29] Laffitte E, Janssens JP, Roux-Lombard P (2009). Tuberculosis screening in patients with psoriasis before antitumour necrosis factor therapy: comparison of an interferon-gamma release assay vs tuberculin skin test. Br J Dermatol..

[30] Lönnroth K, Migliori GB, Abubakar I (2015). Towards tuberculosis elimination: an action framework for low-incidence countries. Eur Respir J.

[31] Field MJ (2001) Tuberculosis in the Workplace. The Occupational Tuberculosis Risk of Health Care Workers. Available from: https://www.ncbi.nlm.nih.gov/books/NBK222462/

[32] Papa A, Mocci G, Bonizzi M (2009). Use of infliximab in particular clinical settings: management based on current evidence. Am J Gastroenterol.

[33] Van Assche G, Lewis JD, Lichtenstein GR (2011). The London position statement of the World Congress of Gastroenterology on Biological Therapy for IBD with the European Crohn's and Colitis Organisation: safety. Am J Gastroenterol.

[34] Athimni S, Slouma M, Dhahri R (2022). Tuberculosis Infection Under Anti-TNF Alpha Treatment. Curr Drug Saf.

[35] Cho SK, Kim D, Won S (2017). Safety of resuming biologic DMARDs in patients who develop tuberculosis after anti-TNF treatment. Semin Arthritis Rheum.

[36] Cantini F, Prignano F, Goletti D (2014). Restarting biologics and management of patients with flares of inflammatory rheumatic disorders or psoriasis during active tuberculosis treatment. J Rheumatol Suppl.

